# P-69. Clinical Characteristics, Antimicrobial Resistance Patterns, and Prognostic Factors of Corynebacterium spp. Bacteremia: A 15-Year Retrospective Study

**DOI:** 10.1093/ofid/ofaf695.298

**Published:** 2026-01-11

**Authors:** T touch Anawilkul, Sorawit Chittrakarn, Siripen Kanchanasuwan, Sarunyou Chusri

**Affiliations:** Prince of songkla university hospital, Hatyai, Songkhla, Thailand; Faculty of Medicine, Prince of Songkla University, hatyai, Songkhla, Thailand; Faculty of Medicine, Prince of Songkla University, hatyai, Songkhla, Thailand; Faculty of Medicine, Prince of Songkla University, hatyai, Songkhla, Thailand

## Abstract

**Background:**

Corynebacterium species are often dismissed as contaminants but can cause serious bloodstream infections. Their clinical significance, resistance, and outcomes remain unclear. This study aimed to identify clinical features, resistance patterns, and predictors of true bacteremia and all-cause mortality.

**Methods:**

We retrospectively reviewed adult patients with Corynebacterium-positive blood cultures at a Thai tertiary hospital (2009–2024). True bacteremia was defined by clinical and microbiological criteria. Species identification using MALDI-TOF MS began in 2017. Data on demographics, clinical features, resistance, and mortality were analyzed. Multivariate logistic regression identified predictors of true bacteremia and 30-day all-cause mortality.

**Results:**

Among 437 patients, 192 (43.9%) had true bacteremia, with *C. striatum* as the predominant species. Predictors of true bacteremia included hematologic malignancy (OR=3.75, 95%CI=1.40–10.73), central venous catheter (CVC) use (OR=2.39, 95%CI=1.49–3.84), hypertension (OR=1.92, 95%CI=1.20–3.08), and chronic kidney disease, non-dialysis (OR=2.83, 95%CI=1.11–7.66). The 30-day mortality rate was 43.8% (84/192). Mortality predictors included hematologic malignancy (OR=4.99, 95%CI=1.34–24.46), aplastic anemia/myelodysplastic syndrome (OR=3.31, 95%CI=1.38–8.20), and CVC use (OR=3.21, 95%CI=1.64–6.41). All isolates were vancomycin-susceptible; however, *C. striatum* had high resistance to ciprofloxacin and clindamycin (1.6%), low susceptibility to imipenem (4.8%), and penicillin (3.3%). Other species showed variable susceptibility.

**Conclusion:**

Nearly half of Corynebacterium isolates from blood cultures represented true bloodstream infections and were associated with high mortality. Hematologic malignancy and CVC use predicted both infection and poor outcomes. Aplastic anemia and myelodysplastic syndrome were also associated with increased 30-day all-cause mortality. Species-level identification and susceptibility testing are essential. Vancomycin remains the most effective empiric therapy.

**Disclosures:**

All Authors: No reported disclosures

